# Rituximab protects against development of atherosclerotic cardiovascular disease after kidney transplantation: a propensity-matched study

**DOI:** 10.1038/s41598-019-52942-8

**Published:** 2019-11-11

**Authors:** Deok Gie Kim, Juhan Lee, Won Jun Seo, Jae Geun Lee, Beom Seok Kim, Myoung Soo Kim, Soon Il Kim, Yu Seun Kim, Kyu Ha Huh

**Affiliations:** 1 Department of Surgery, Yonsei University Wonju College of Medicine, Wonju, Republic of Korea; 2 Department of Surgery, Yonsei University College of Medicine, Seoul, Republic of Korea; 3 Department of Surgery, Korea University College of Medicine, Seoul, Republic of Korea; 4 Department of Internal Medicine, Yonsei University College of Medicine, Seoul, Republic of Korea; 5 The Research Institute for Transplantation, Yonsei University College of Medicine, Seoul, Republic of Korea

**Keywords:** Cardiovascular diseases, Nephrons

## Abstract

Recent studies have implicated B cells in atherosclerosis and have verified the atheroprotective effect of rituximab. Rituximab is widely used for desensitization in ABO-incompatible or crossmatch-positive kidney transplantation (KT). Using a single-center KT database, we performed propensity-matched analysis to investigate the association between rituximab and posttransplant atherosclerotic cardiovascular disease (ASCVD). Among 1299 eligible patients, 239 given rituximab induction were matched with 401 controls in a 1:2 propensity score matching process. The cumulative rate of ASCVD during 8 years of follow-up was significantly lower in rituximab-treated patients, compared with matched controls (3.7% vs. 11.2%; *P* = 0.012). However, all-cause mortality did not differ by group (2.9% vs. 4%; *P* = 0.943). In multivariable Cox analysis, rituximab proved independently protective of ASCVD (hazard ratio = 0.34, 95% confidence interval: 0.14–0.83). The lower risk of ASCVD seen with rituximab induction reached significance only in patient subsets of diabetes mellitus, pretransplant dialysis, or older age (>50 years). Rituximab induction confers a lower risk of ASCVD during the posttransplant period. This atheroprotective effect appears particularly beneficial in patients whose risk of ASCVD is heightened.

## Introduction

Cardiovascular disease (CVD) is the leading cause of death after kidney transplantation (KT)^1^, and the incidence of CVD is higher in KT recipients than in the general population^2^. Numerous studies reported that the risk of CVD is elevated after KT because of non-traditional risk factors such as immunologic alteration and the various medications related to the transplantation surgery^3–9^. Atherosclerosis is an underlying chronic inflammatory condition in CVD, and the role of the immune system in the development of atherosclerosis is well descried in the literature^10^. B lymphocytes were considered to have a protective effect against atherosclerosis until nearly 2010^11^. However, recent experimental studies revealed opposing roles of different B cell subsets, atheroprotective B1 cells and atherogenic B2 cells^12–16^. Those studies also demonstrated reduced atherosclerotic plaques in atherosclerosis-prone mice treated with rituximab, a B cell-depleting anti-CD20 monoclonal antibody. The potential of B cell targeting as a treatment for atherogenesis has been suggested^17^, although heretofore there was no comparative human study of B cell depletion and atherosclerotic CVD (ASCVD).

Rituximab was introduced as an effective alternative to splenectomy for desensitization of patients requiring KT^18^. Given the excellent outcomes reported in ABO-incompatible and crossmatch-positive KT^19,20^, rituximab use in the realm of KT is increasing. Herein, we sought to explore the effects of rituximab induction on ASCVD in the aftermath of KT through propensity-matched analysis.

## Results

### Descriptions of entire cohort

For the 1299 eligible patients, mean age was 45.3 ± 11.3 years, and there were 761 men (58.6%). Nine hundred forty-five patients (72.7%) underwent living donor kidney transplantation (LDKT), and in 126 (9.7%), retransplantation took place. One hundred ten grafts were lost including 46 deaths (7 as cardiovascular deaths) and 77 ASCVDs occurred during a mean follow-up of 82.3 ± 35.5 months. Rituximab was administered preoperatively to 245 patients (18.9%; 117 ABO incompatibility, 43 crossmatch positivity, 13 simultaneous ABO incompatibility and crossmatch positivity, and 72 high reactivity to panel antibodies). Among patients treated with rituximab, 167 (68.1%) were given standard doses (375 mg/m^2^), and 78 (31.9%) received reduced doses (200 mg).

### Baseline characteristics before and after matching

Table 1 shows baseline characteristics of rituximab and control group patients. Before matching, there were differences in some variables, such as sex, deceased donor, duration of dialysis, retransplantation, tacrolimus use, pretransplant diabetes melitus (DM), baseline Low-density lipoprotein (LDL), biopsy-proven acute rejection (BPAR) within 1 year, and estimated glomerular filtration rate (eGFR) at 1 month. In 1:2 propensity score matching, 239 patients given rituximab were matched with 401 controls. Most variables (except BPAR within 1 year) were then balanced within the two groups (see *Statistical analysis*).

**Table 1:** Baseline characteristics before and after propensity score matching.

Variables	Before matching			After matching		
	Rituximab (n = 245)	Control (n = 1054)	*P*	Rituximab (n = 239)	Control (n = 401)	*P*
Age (years)	46.1 ± 10.5	45.1 ± 11.5	0.210	46.0 ± 10.6	44.6 ± 12.2	0.144
Sex, males	116 (47.3%)	645 (61.2%)	<0.001	115 (48.1%)	207 (51.6%)	0.391
Body mass index (kg/m^2^)	22.3 ± 3.4	22.5 ± 3.3	0.298	22.3 ± 3.4	22.3 ± 3.5	0.754
Deceased donor	20 (8.2%)	334 (31.7%)	<0.001	20 (8.4%)	40 (10.0%)	0.500
Dialysis duration (months)	4 (27)	13 (73)	<0.001	4 (28)	5 (28)	0.429
Retransplantation	39 (15.9%)	87 (8.3%)	<0.001	33 (13.8%)	38 (9.5%)	0.091
Use of tacrolimus (vs. cyclosporin)	237 (96.7%)	776 (73.6%)	<0.001	231 (96.7%)	384 (95.8%)	0.573
Pretrasnplant alcohol use	47 (19.2%)	184 (17.5%)	0.524	47 (19.7%)	61 (15.2%)	0.146
Pretransplant smoking	47 (19.2%)	215 (20.4%)	0.669	47 (19.7%)	77 (19.2%)	0.886
Pretransplant DM	71 (29.0%)	193 (18.3%)	<0.001	66 (27.6%)	105 (26.2%)	0.692
Pretransplant ASCVD history	19 (7.8%)	71 (6.7%)	0.572	18 (7.5%)	31 (7.7%)	0.927
SBP ≥ 140 mm Hg	111 (50.9%)	568 (57.5%)	0.074	109 (51.4%)	214 (56.3%)	0.251
DBP ≥ 90 mm Hg	84 (38.7%)	436 (44.2%)	0.138	83 (39.3%)	168 (44.3%)	0.240
Total cholesterol ≥ 240 (mg/dL)	6 (2.4%)	30 (2.8%)	0.733	6 (2.5%)	8 (2.0%)	0.666
LDL cholesterol ≥ 100 (mg/dL)	39 (15.9%)	323 (30.7%)	<0.001	39 (16.3%)	76 (19.0%)	0.401
Use of statin	51 (20.8%)	214 (20.3%)	0.858	48 (20.1%)	90 (22.4%)	0.482
Biopsy-proven acute rejection within 1 year	64 (26.1%)	130 (12.3%)	<0.001	62 (25.9%)	41 (10.2%)	<0.001
eGFR^a^ at 1 month (mL/min)	66.4 ± 24.6	62.9 ± 21.3	0.042	65.7 ± 24.3	65.1 ± 20.6	0.715

^a^Calculated using Chronic Kidney Disease Epidemiology (CKD-EPI) formula.

ASCVD, atherosclerotic cardiovascular disease; DBP, diastolic blood pressure; DM, diabetes mellitus; eGFR, estimated glomerular filtration rate; KT, kidney transplantation; LDL, low density lipoprotein; SBP, systolic blood pressure.

### ASCVD and all-cause death in matched cohort

**Figure 1:**
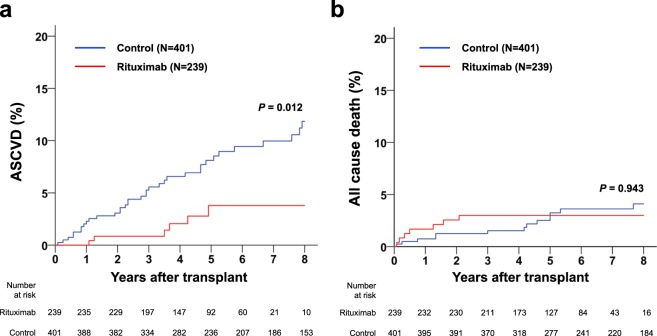
Comparison of (**a**) ASCVD and (**b**) all cause death after KT by rituximab induction in the matched cohort. KT, kidney transplantation; ASCVD, atherosclerotic cardiovascular disease.

In the matched cohort, patients undergoing rituximab induction experienced only 6 ASCVD events within 8 years after KT (1 fatal myocardial infarction [MI] and 5 percutaneous coronary revascularizations). However, 35 ASCD events were recorded in the control group (3 fatal MIs, 1 nonfatal MI, 8 percutaneous coronary revascularizations, 4 coronary artery bypass surgeries, 8 acute cerebral infarctions, and 11 peripheral artery revascularizations). The cumulative rate of ASCVD was significantly lower in rituximab-treated patients than in controls (3.7% vs. 11.2%, *P* = 0.012; Fig. 1a). Seven deaths occurred in the rituximab group, including one cardiovascular death. Among 13 posttransplant deaths in the control group, three were from cardiovascular causes. During the 8-year follow-up period, rituximab-treated patients and controls showed no significant difference in all-cause death (2.9% vs. 4%, *P* = 0.943; Fig. 1b). ASCVD events and causes of death in both groups are detailed in Table 2.

**Table 2:** The first ASCVD events and cause of mortality over 8 years in the matched cohort.

	Rituximab (n = 239)	Control (n = 401)
**ASCVD events**		
Total	6	35
Fatal MI	1	3
Non-fatal MI	0	1
Percutaneous coronary revascularization	5	8
Coronary artery bypass surgery	0	4
Acute cerebral infarction	0	8
Peripheral artery revascularization	0	11
**Cause of mortality**		
Total	7	13
Cardiovascular	1	3
Infection	3	9
Gastrointestinal bleeding	1	0
Malignancy	0	1
Liver disease	1	0
Unknown	1	0

ASCVD, atherosclerotic cardiovascular disease; MI, myocardial infarction.

### Serial comparison of blood pressure, renal function, and LDL

**Figure 2:**
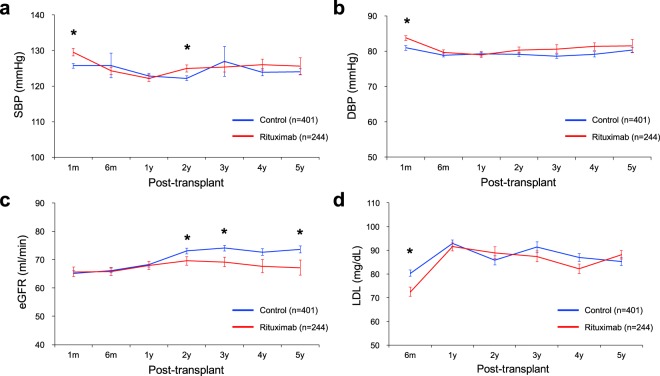
Serial comparison of (**a**) SBP, (**b**) DBP, (**c**) eGFR and (**d**) LDL between the rituximab group and the matched controls. *P < 0.05, DBP, diastolic blood pressure; eGFR, estimated glomerular filtration rate; LDL, low density lipoprotein; SBP, systolic blood pressure.

Figure 2 depicts changes over time in several variables known to be CVD risk factors. Although systolic blood pressure (SBP) and diastolic blood pressure (DBP) were higher in treated patients (vs controls) 1 month after transplantation, both levels appeared similar thereafter at most points in time. eGFR did not differ by group up to 1 year posttransplantation, surging higher in controls than in rituximab-treated patients at Years 2 and 3. LDL was higher in controls at 6 months only, approaching levels similar to treated patients for the duration of follow-up.

### Cox proportional hazard analysis for ASCVD

In multivariable Cox analysis of matched cohort data (Table 3), rituximab emerged as independently protective of ASCVD developing after KT (hazard ratio [HR] = 0.34, 95% confidence interval [CI]: 0.14–0.83). Other risk factors for ASCVD were maleness (HR = 2.14, 95% CI: 1.01–4.52), duration of dialysis (HR = 1.07, 95% CI: 1.03–1.11 [per 6 months]), retransplantation (HR = 2.90, 95% CI: 1.34–6.27), pretransplant DM (HR = 3.68, 95% CI: 1.71–7.88), and pretransplant ASCVD history (HR = 4.92, 95% CI: 2.42–10.03). BPAR within 1 year, which differed in rituximab and control groups, was not a risk factor (HR = 0.95, 95% CI: 0.40–2.25). LDL cholesterol, statin use, blood pressure, and eGFR at 1 month were not associated with posttransplant ASCVD.

**Table 3:** Risk factors associated with ASCVD in matched cohort.

Variables	Univariable Cox		Multivariable Cox^a^	
	HR (95% CI)	*P*	HR (95% CI)	*P*
Age (per 5 years)	1.32 (1.15–1.53)	<0.001		
Sex, male	3.25 (1.59–6.63)	0.001	2.14 (1.01–4.52)	0.047
Body mass index ≥ 25 kg/m^2^	1.21 (0.58–2.53)	0.618		
Deceased donor	1.20 (0.43–3.36)	0.735		
Dialysis duration (per 6 months)	1.05 (1.02–1.09)	0.002	1.07 (1.03–1.11)	<0.001
Retransplantation	2.42 (1.15–5.07)	0.019	2.90 (1.34–6.27)	0.007
Pretrasnplant alcohol use	1.37 (0.63–2.99)	0.423		
Pretransplant smoking	2.11 (1.07–4.15)	0.031		
Pretransplant DM	5.98 (3.15–11.33)	<0.001	3.68 (1.71–7.88)	0.001
Pretransplant ASCVD history	9.29 (4.94–17.47)	<0.001	4.92 (2.42–10.03)	<0.001
Baseline SBP ≥ 140 mm Hg	1.15 (0.61–2.16)	0.672		
Baseline DBP ≥ 90 mm Hg	0.62 (0.31–1.23)	0.169		
LDL cholesterol ≥ 100 mg/dL	1.24 (0.59–2.60)	0.565		
Use of statin	1.57 (0.80–3.09)	0.187		
Biopsy proven acute rejection within 1 year	0.95 (0.40–2.25)	0.902		
eGFR^b^ at 1 month (per 10 mL/min)	0.97 (0.83–1.12)	0.633		
Rituximab induction	0.34 (0.14–0.82)	0.017	0.34 (0.14–0.83)	0.017

^a^Multivariable Cox analysis was performed by backward stepwise selection with *P*-value threshold 0.05.

^b^Calculated using Chronic Kidney Disease Epidemiology (CKD-EPI) formula.

ASCVD, atherosclerotic cardiovascular disease; CI, confidence interval; DBP, diastolic blood pressure; DM, diabetes mellitus; eGFR, estimated glomerular filtration rate; KT, kidney transplantation; LDL, low density lipoprotein; SBP, systolic blood pressure.

### Subgroup analysis

**Figure 3:**
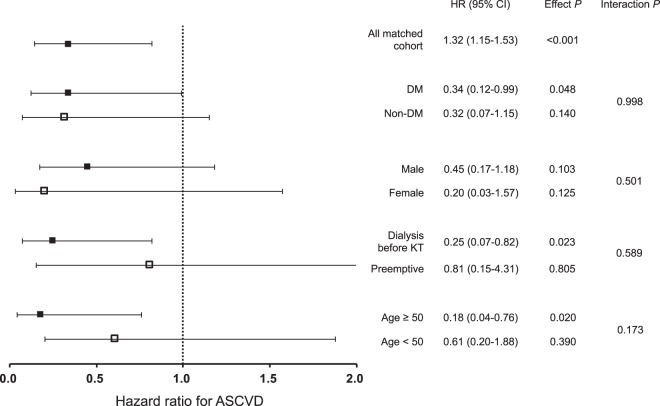
Hazard ratios for posttransplant ASCVD in clinical subgroups treated with rituximab. From matched cohort, subjects were stratified by DM, sex, pretransplant dialysis and 50 years of age. CI, confidence interval; DM, diabetes mellitus; HR, hazard ratio; KT, kidney transplantation; ASCVD, atherosclerotic cardiovascular disease.

Subjects of the matched cohort were stratified by pretransplant DM, sex, pretransplant dialysis, and age ≥ 50 years, followed by analyzing HRs of rituximab treatment for ASCVD in each subgroup (Fig. 3). There was no apparent interaction between any subgroup and rituximab treatment. HRs of patients with and without DM were 0.34 (95% CI: 0.12–0.99; *P* = 0.048) and 0.32 (95% CI: 0.07–1.15; *P* = 0.140), respectively. Respective HRs of male and female patients were 0.45 (95% CI: 0.17–1.18; *P* = 0.103) and 0.20 (95% CI: 0.03–1.57; *P* = 0.125), neither showing significance. In the presence and absence of dialysis prior to KT, HRs were 0.25 (95% CI: 0.07–0.82; *P* = 0.023) and 0.81 (95% CI: 0.15–4.31; *P* = 0.805), respectively; and HRs of patients beyond or less than 50 years old were 0.61 (95% CI: 0.20–1.88; *P* = 0.020) and 0.61 (95% CI: 0.20–1.88; *P* = 0.390), respectively.

## Discussion

In the propensity-matched analysis herein, rituximab induction significantly reduced the incidence of ASCVD developing after KT. Prior *in vivo* studies demonstrated that the use of anti-B-cell agents could be an effective therapeutic approach for the treatment and prevention of atherosclerosis^15^. Few studies have investigated the atheroprotective effect of rituximab using clinical data. Hsue *et al*.^21^ reported that rituximab improved endothelial function and reduced inflammation in patients with rheumatoid arthritis; however, they did not show a difference in ASCVD based on rituximab treatment.

There has been concern about cardiac complications after the use of rituximab to treat hematologic or rheumatologic diseases^22–24^. However, acute or delayed cardiac toxicity was suggested to be a result of cytokine release related to the infusion-related reaction rather than an aggravation of atherosclerosis. In addition, several randomized controlled trials showed that there was no difference in cardiac adverse events between patients with or without rituximab treatment for lymphoma and rheumatoid arthritis^25,26^.

In the field of renal transplantation, there was only one report on rituximab and CVD. Tyden *et al*.^27^ reported higher mortality from MI in patients treated with rituximab than in controls. That result came from a 3-year follow-up of a randomized control trial, but the number of participants was small, and there was no information about CVD risk factors. Also, the incidence of fatal MI was higher (about 10% during just 3 years of follow-up) compared with previous reports^4,5,28^. The high rate of cardiac mortality might be attributable to other CVD-related patient factors rather than to an adverse effect of rituximab. In fact, another recently conducted randomized controlled trial has established a safety profile for rituximab in terms of serious adverse events in KT^29^.

Rituximab is a monoclonal antibody that globally destroys CD 20-positive B cells. However, the aforementioned experimental evidence demonstrated that rituximab reduced atherosclerosis. That atheroprotective effect might come from the fact that atherogenic B2 cells are a major population of the adult human B cell repertoire^30^. Another hypothesis is that because the peritoneal cavity provides rituximab resistance, resident B1 cells may be spared^31^. Although the mechanism of the atheroprotective effect of rituximab is not yet known in humans, our results provide an important hint for investigating the role of B cell subsets in atherogenesis.

We found that the incidence of ASCVD was relatively low in our study population. Previous large-volume studies in Western populations have cited CVD rates of ~15% during 4–5 years of follow-up after KT^8,32^, whereas that rate was only 6% during entire follow-up in our study. The difference might be due to dietary and lifestyle differences as well as the lower incidence of pretransplant ASCVD observed in Korea, compared to Western countries. Furthermore, more than 20% of patients were using statin before KT, so the mean LDL level (84.4 mg/dL) in our study exceeded by the mean (100 mg/dL) determined in Western studies. The relatively low incidence of CVD in Korean KT patients was also observed in another Korean study^33^ and in a previous international cohort^4^.

Anti-B-cell agents, including rituximab and B cell activating factor receptor antibody (belimumab), have been suggested as possible anti-atherogenic treatments^17^, but there has not yet been a clinical study. In a situation where the number of KTs with prior desensitization is increasing^34^, especially in Korea where LDKT prevails^35,36^, our results provide clinical evidence to help understand the role of rituximab in ASCVD development.

Through subgroup analysis, we extended our investigation to various CVD risk factors and their influence on rituximab atheroprotection. Rituximab proved significantly protective in specific clinical subsets, namely DM, pretransplant dialysis, and older age ( ≥ 50 years); but no HRs proved significant in the absence of these factors. In addition, gender of KT recipients had no apparent impact on ASCVD in rituximab-treated patients. Adjusted HRs were unobtainable for subgroups, given the low event totals. However, assessment of matched subgroups revealed greater rituximab atheroprotection in patients at higher risk of ASCVD. A broad multicenter study, perhaps utilizing a national registry, would likely yield more conclusive results.

Despite our propensity-matched analysis, retrospective feature is still limitation of our study for which the results should be cautiously interpreted. Also, single center design is another major limitation. Prospective and multicenter study should be needed.

In conclusion, this study has demonstrated that rituximab induction reduces the rate of ASCVD during posttransplant periods. Furthermore, the atheroprotective effect of rituximab appears more advantageous for some KT recipients with heightened risks of ASCVD.

## Methods

### Patient selection

**Figure 4:**
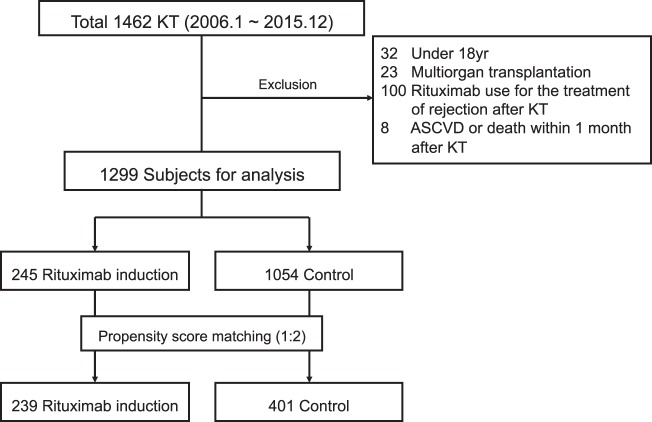
Study population. KT, kidney transplantation; ASCVD, atherosclerotic cardiovascular disease.

We conducted a retrospective study, accessing a database of 1,462 patients who underwent KT at Severance Hospital, Seoul, Korea, between January 2006 and December 2015. Exclusion criteria were as follows: (1) age < 18 years at time of KT, (2) multiorgan transplantation, (3) rituximab use in treating antibody-mediated rejection after KT, and (4) acute coronary disease or patient death within 1 month after KT (2 fatal MIs, 2 nonfatal MIs, 2 death from cerebrovascular accidents, 1 non-cardiovascular death, 1 peripheral artery revascularization). Ultimately, 1299 patients qualified for analysis (Fig. 4).

### Data collection

We collected data on baseline characteristics of recipients and donors, lipid profiles, pretransplant use of statins, and BPAR within 1 year posttransplantation. SBP, DBP and eGFR were checked every 3 months. LDL concentrations were checked every 6 months for 1 year posttransplantation and annually thereafter. During posttransplant periods, we checked electrocardiography at least yearly. Patients with cardiac symptoms or electrocardiographic abnormalities were referred to a cardiologist for further evaluation. Those undergoing coronary revascularization were routinely followed by coronary angiography every 1–2 years, screening for restenosis or newly developed lesions. If suspected, patients were evaluated for acute cerebral infarction and peripheral arterial occlusive disease. We defined ASCVD as any of the following: fatal or nonfatal MI, coronary revascularization (by percutaneous intervention or surgery), acute cerebral infarction, or revascularization of peripheral arterial occlusion (by percutaneous intervention or surgery).

### Rituximab use in desensitization protocol

Indications for rituximab induction were ABO-incompatible or crossmatch-positive KT (living donors, both instances) and ≥ 50% panel reactive antibodies prior to KT (living or deceased donors). In the event of LDKT, rituximab was given within 7–14 days in advance, delivering a single dose of 375 mg/m^2^. As of August 2013, rituximab dosing for ABO-incompatible LDKT was reduced to 200 mg if baseline anti-donor isoagglutinin IgG titers were ≤1:128^37^. For deceased donor kidney transplantation, 200 mg of rituximab was given prior to operations as indicated. Standard regimens used at our institution for preoperative desensitization and postoperative immunosuppression are well described elsewhere^37–39^.

### Study outcomes

We compared composite ASCVD and all-cause mortality by rituximab induction in the matched cohort. Because the longest follow-up period in rituximab-treated patients was 102 months, we limited such comparisons to 8 years after KT. We also conducted subgroup analyses, examining various patient characteristics (DM, sex, required pretransplant dialysis, and age ≥ 50 years) to assess the potential influence of each in relation to ASCVD and rituximab use.

### Statistical analysis

Propensity matching was undertaken to coordinate baseline characteristics of the two groups (with or without rituximab induction). Subsequent scores generated via logistic regression analysis included the following covariates, which differed for above groups or were known risk factors for CVD in KT recipients^4,28^: age, sex, deceased donor, duration of dialysis, retransplantation, tacrolimus use, smoking, pretransplant DM, pretransplant ASCVD history, baseline LDL, and eGFR at 1 month. BPAR was not matched, because rituximab-treated patients had high risk for rejection by nature, and because BPAR had no impact on development of ASCVD in our entire cohort. Using the nearest neighbor method, we matched patients given rituximab to controls at 1:2 without replacement, setting the caliper at 0.25 width. All assessments were achieved using R freeware v3.5.0 (R Foundation for Statistical Computing, Vienna, Austria) with *MatchIt* package^40^.

In comparing baseline characteristics of both groups before and after matching, data were expressed as mean ± standard deviation or median (interquartile range) for continuous variables and numbers (%) for categorical variables. Student’s *t*-test, Mann-Whitney U test, or chi square test was applied as needed for group comparisons. We used Kaplan-Meier analysis to estimate differences in probabilities of ASCVD and all-cause mortality according to rituximab induction and calculated *P*-values by log-rank test. For estimates of ASCVD, death from causes other than ASCVD served for censoring.

In the matched cohort, we invoked Cox proportional-hazards regression analysis to assess the relation between rituximab induction and posttransplant ASCVD. Multivariable Cox regression entailed backward stepwise selection, based on covariates with *P*-values < 0.05 in univariable analysis. Patients were further stratified by known CVD risk factors such as DM, sex, pretransplant dialysis, and age ≥ 50. To retain the homogeneity of rituximab and control groups, only matched pairs with or without specified variables qualified for subgroup analysis. Small ASCVD events in each subgroup prohibited multivariable Cox regression, so we presented only unadjusted HR of rituximab induction in subgroup analysis. All comparative and survival analyses relied on standard software (SPSS v23.0; IBM, Armonk, NY, USA), setting statistical significance at *P* < 0.05.

### Ethics statement

This study was conducted according to the principles of the Declaration of Helsinki and the Declaration of Istanbul. It was also approved by the Institutional Review Board of Severance Hospital, Yonsei University Health System (IRB No.: 4-2018-0355) with exemption for the informed consent due to the retrospective feature of this study.

## References

[1] 2017 USRDS annual data report, Vol 2 - ESRD in the United States, Chapter 5: Mortality. *Am J Kidney Dis* **71**, S337–S350, 10.1053/j.ajkd.2018.01.018 (2018).

[2] Sarnak MJ (2003). Kidney disease as a risk factor for development of cardiovascular disease: a statement from the American Heart Association Councils on Kidney in Cardiovascular Disease, High Blood Pressure Research, Clinical Cardiology, and Epidemiology and Prevention. Circulation.

[3] Stoumpos S, Jardine AG, Mark PB (2015). Cardiovascular morbidity and mortality after kidney transplantation. Transpl Int.

[4] Israni AK (2010). Predicting coronary heart disease after kidney transplantation: Patient Outcomes in Renal Transplantation (PORT) Study. Am J Transplant.

[5] Vanrenterghem YF (2008). Risk factors for cardiovascular events after successful renal transplantation. Transplantation.

[6] Marcen R (2006). Cardiovascular risk factors in renal transplantation–current controversies. Nephrol Dial Transplant.

[7] Jardine AG (2005). Cardiovascular risk and renal transplantation: post hoc analyses of the Assessment of Lescol in Renal Transplantation (ALERT) Study. Am J Kidney Dis.

[8] Holdaas H (2005). Long-term cardiac outcomes in renal transplant recipients receiving fluvastatin: the ALERT extension study. Am J Transplant.

[9] Holdaas H (2003). Effect of fluvastatin on cardiac outcomes in renal transplant recipients: a multicentre, randomised, placebo-controlled trial. Lancet.

[10] Moriya J (2018). Critical roles of inflammation in atherosclerosis. J Cardiol.

[11] Caligiuri G, Nicoletti A, Poirier B, Hansson GK (2002). Protective immunity against atherosclerosis carried by B cells of hypercholesterolemic mice. J Clin Invest.

[12] Kyaw T (2010). Conventional B2 B cell depletion ameliorates whereas its adoptive transfer aggravates atherosclerosis. J Immunol.

[13] Ait-Oufella H (2010). B cell depletion reduces the development of atherosclerosis in mice. J Exp Med.

[14] Kyaw T (2011). B1a B lymphocytes are atheroprotective by secreting natural IgM that increases IgM deposits and reduces necrotic cores in atherosclerotic lesions. Circ Res.

[15] Tsiantoulas D, Diehl CJ, Witztum JL, Binder CJ (2014). B cells and humoral immunity in atherosclerosis. Circ Res.

[16] Kyaw T, Tipping P, Bobik A, Toh BH (2017). Opposing roles of B lymphocyte subsets in atherosclerosis. Autoimmunity.

[17] Tsiantoulas D, Sage AP, Mallat Z, Binder CJ (2015). Targeting B cells in atherosclerosis: closing the gap from bench to bedside. Arterioscler Thromb Vasc Biol.

[18] Tanabe K (2007). Japanese experience of ABO-incompatible living kidney transplantation. Transplantation.

[19] Orandi BJ (2016). Survival Benefit with Kidney Transplants from HLA-Incompatible Live Donors. N Engl J Med.

[20] Opelz G (2015). Three-year outcomes following 1420 ABO-incompatible living-donor kidney transplants performed after ABO antibody reduction: results from 101 centers. Transplantation.

[21] Hsue PY (2014). Depletion of B-cells with rituximab improves endothelial function and reduces inflammation among individuals with rheumatoid arthritis. J Am Heart Assoc.

[22] Foran JM (2000). European phase II study of rituximab (chimeric anti-CD20 monoclonal antibody) for patients with newly diagnosed mantle-cell lymphoma and previously treated mantle-cell lymphoma, immunocytoma, and small B-cell lymphocytic lymphoma. J Clin Oncol.

[23] Armitage JD, Montero C, Benner A, Armitage JO, Bociek G (2008). Acute coronary syndromes complicating the first infusion of rituximab. Clin Lymphoma Myeloma.

[24] Mehrpooya M, Vaseghi G, Eshraghi A, Eslami N (2016). Delayed Myocardial Infarction Associated With Rituximab Infusion: A Case Report and Literature Review. Am J Ther.

[25] Emery P (2010). Efficacy and safety of different doses and retreatment of rituximab: a randomised, placebo-controlled trial in patients who are biological naive with active rheumatoid arthritis and an inadequate response to methotrexate (Study Evaluating Rituximab’s Efficacy in MTX iNadequate rEsponders (SERENE). Ann Rheum Dis.

[26] Provencio M (2009). A prospective study of left ventricle function after treatment with rapid-infusion rituximab in patients with non-Hodgkin lymphoma. Leuk Lymphoma.

[27] Tyden G, Ekberg H, Tufveson G, Mjornstedt L (2012). A randomized, double-blind, placebo-controlled study of single dose rituximab as induction in renal transplantation: a 3-year follow-up. Transplantation.

[28] Soveri I (2012). A cardiovascular risk calculator for renal transplant recipients. Transplantation.

[29] van den Hoogen MW (2015). Rituximab as induction therapy after renal transplantation: a randomized, double-blind, placebo-controlled study of efficacy and safety. Am J Transplant.

[30] Sagaert X, De Wolf-Peeters C (2003). Classification of B-cells according to their differentiation status, their micro-anatomical localisation and their developmental lineage. Immunology Letters.

[31] Hamaguchi Y (2005). The peritoneal cavity provides a protective niche for B1 and conventional B lymphocytes during anti-CD20 immunotherapy in mice. J Immunol.

[32] Carpenter MA (2014). BP, cardiovascular disease, and death in the Folic Acid for Vascular Outcome Reduction in Transplantation trial. J Am Soc Nephrol.

[33] Jeong JC (2010). Cardiovascular diseases after kidney transplantation in Korea. J Korean Med Sci.

[34] Morath C, Zeier M, Dohler B, Opelz G, Susal C (2017). ABO-Incompatible Kidney Transplantation. Front Immunol.

[35] Ko EJ, Yu JH, Yang CW, Chung BH, Korean Organ Transplantation Registry Study, G. (2017). Clinical outcomes of ABO- and HLA-incompatible kidney transplantation: a nationwide cohort study. Transpl Int.

[36] Kwon H (2016). Analysis of 4000 kidney transplantations in a single center: Across immunological barriers. Medicine (Baltimore).

[37] Lee J (2016). The effect of rituximab dose on infectious complications in ABO-incompatible kidney transplantation. Nephrol Dial Transplant.

[38] Song SH (2017). Successful launch of an ABO-incompatible kidney transplantation program to overcome the shortage of compatible living donors: experience at a single center. Clin Nephrol.

[39] Huh KH (2012). Renal transplantation in sensitized recipients with positive luminex and negative CDC (complement-dependent cytotoxicity) crossmatches. Transpl Int.

[40] Ho DE, Imai K, King G, Stuart EA (2007). Matching as Nonparametric Preprocessing for Reducing Model Dependence in Parametric Causal Inference. Political Analysis.

